# Construction and Validation of a Newly Prognostic Signature for CRISPR-Cas9-Based Cancer Dependency Map Genes in Breast Cancer

**DOI:** 10.1155/2022/4566577

**Published:** 2022-01-19

**Authors:** Xin Yan, Sai-Nan You, Yan Chen, Ke Qian

**Affiliations:** ^1^Liyang People's Hospital, Liyang 213399, China; ^2^Suqian Hospital of Nanjing Drum Tower Hospital Group, Suqian 223800, China; ^3^Jiangsu Province Geriatric Hospital, Nanjing 210029, China

## Abstract

Cancer Dependency Map (CDM) genes comprise an extensive series of genome-scale RNAi-based loss-of-function tests; hence, it served as a method based on the CRISPR-Cas9 technique that could assist scientists in investigating potential gene functions. These CDM genes have a role in tumor cell survival and proliferation, suggesting that they may be used as new therapeutic targets for some malignant tumors. So far, there have been less research on the involvement of CDM genes in breast cancer, and only a tiny percentage of CDM genes have been studied. In this study, information of patients with breast cancer was extracted from The Cancer Genome Atlas (TCGA), from which differentially expressed CDM genes in breast cancer were determined. A variety of bioinformatics techniques were used to assess the functions and prognostic relevance of these confirmed CDM genes. In all, 290 CDM genes were found differentially expressed. Six CDM genes (SRF, RAD51, PMF1, EXOSC3, EXOC1, and TSEN54) were found to be associated with the prognosis of breast cancer samples. Based on the expression of the identified CDM genes and their coefficients, a prognosis model was constructed for prediction, according to which patients with breast cancer were separated into two risk groups. Those with high risk had substantially poorer overall survival (OS) than patients in the other risk group in the TCGA training set, TCGA testing set, and an external cohort from Gene Expression Omnibus (GEO) database. The area under the receiver operating characteristic (ROC) curve for this prognostic signature was, respectively, 0.717 and 0.635 for TCGA training and testing sets, demonstrating its reliability in predicting the prognosis of patients with breast cancer. We next created a nomogram using the six CDM genes discovered to create a therapeutically useful model. The Human Protein Atlas database was used to acquire all immunohistochemistry staining images of the discovered CDM genes. The proportions of tumor-infiltrating immune cells, as well as the expression levels of checkpoint genes, varied substantially between the two risk groups, according to the analyses of immune response. In conclusion, the findings of this research may aid in the understanding of the prognostic value and biological roles of CDM genes in breast cancer.

## 1. Introduction

Breast cancer is one of the most prevalent malignancies in women [1]. Meanwhile, male breast cancer accounts for approximately 1% of all breast cancer cases globally, and clinical data suggest that it has been on the increase in recent years [2]. Breast cancer screening has been shown to decrease mortality from the disease [3]. Patients with breast cancer have a five-year survival rate of almost 98% if the illness is identified early [4]. Although the survival rate for breast cancer patients has improved, the survival rate for advanced-stage patients remains low, particularly for patients with locally advanced breast cancer [5], which affects up to 20% of patients with breast cancer [6]. Hence, understanding the molecular processes that cause breast cancer remains an important challenge for us to develop new treatments [7].

The Broad Institute and the Dana-Farber Cancer Institute started a project called “Defining a Cancer Dependency Map” to find genes that encouraged cancer cell development [8]. This significant research demonstrated the significance of certain genes in the development and proliferation of cancer cells. These certain genes may be used as targets for the development of new targeted medicines. More than 500 human cancer cell lines that were often used to investigate the impact of shutting off specific genes on proliferation and growth for their ability of growing indefinitely were examined in this research. In detail, cancer cell lines were transformed with a lentiviral vector expressing Cas9 nuclease under cystatin selection (pXPR-311Cas9) [9]. Cas9 activity assays were performed on each Cas9-expressing cell line to characterize the effect of CRISPR/Cas9 on these cell lines. Cell lines that detected Cas9 activity below 45% were not eligible for further screening. Although the majority of these identified certain genes were cancer-specific, approximately 10% of them were shown to be involved in various malignant tumors, indicating that they had important biological roles. This research also found that studying gene activity patterns rather than concentrating on whether a particular gene was faulty was the best method to anticipate this dependency [10]. More relevant genes were identified and uploaded to the website (https://depmap.org/portal/) as the study progressed and real-time updates were made [9]. A total of 1246 CDM genes were included in the study in the most recent update of the dataset, and they were shown to be frequent and important in the incidence and development of different malignancies.

In this research, breast cancer data from TCGA was downloaded, and differentially expressed CDM genes were identified, as well as their potential roles and processes. A CDM gene-based prognosis signature was then created and further validated, based on which, some CDM genes may be utilized as possible prognostic biomarkers.

## 2. Materials and Methods

### 2.1. Data Collection and Processing

RNA-sequencing and clinical feature information of breast cancer were all downloaded online from a database named The Cancer Genome Atlas (TCGA, https://portal.gdc.cancer.gov/). A total of 1,246 CDM genes were acquired from a project named Cancer Dependency Map online (https://depmap.org/portal/) [9]. CDM genes with FDR adjusted *P* values <0.05 along with the log2|fold change| values >0.5 were identified as candidate ones since they were significantly differentially expressed between breast cancer patients and normal samples in TCGA training set using packages of R software (version 4.0.5). Volcano plots and heatmaps were, respectively, visualized with the “ggplot2” [11] and “pheatmap” [12] packages in R software. The TCGA testing set was used to validate the results from training set. An external validation dataset of breast cancer, which included transcription profiles and clinical information, were downloaded from the Gene Expression Omnibus (GEO) database (https://www.ncbi.nlm.nih.gov/geo/query/acc.cgi?acc=GSE53031).

### 2.2. Enrichment and Pathway Analyses

To explore the function of identified prognosis-related CDM genes, function and pathway enrichment analyses were carried out. Pearson correlation test was also used to assess those candidate CDM genes. Genes were further classified via the package named “clusterProfiler” [13] in R software according to the projection of GO [14] (http://www.geneontology.org/) or KEGG [15] (http://www.genome.jp/kegg/pathway.html) pathways at a specific level. Of course, GO terms or KEGG pathway with a corrected-*P* < 0.05 were considered as significant enrichment.

### 2.3. Construction and Validation of a CDM Gene-Based Risk Model

The samples in the breast cancer cohort from TCGA were randomly separated into two sets: a training set containing 524 samples and a testing set containing 522 samples. Univariate Cox regression analysis was carried out on those identified prognosis-related CDM genes using R software. The log-rank test was performed to analyze the statistical significance. Multivariate Cox regression analysis was further carried out on the genes chosen by univariate Cox regression analysis to identify ones with independently prognostic value. The six CDM genes that were finally identified by multivariate Cox regression analysis were then used to construct a risk model using their expression levels and coefficient values. The risk score (RS) was calculated for each sample in TCGA training set, the median one of which was chosen as cut-off. According to the cut-off value, patients with breast cancer were divided into the high-risk group or low-risk group. Overall survival (OS) and receiver operating characteristic (ROC) curve analyses were carried out by packages in R software to evaluate the predictive value of the risk model in breast cancer, which was further confirmed by the TCGA testing set. Besides, we established a nomogram based on the constructed risk model and independent clinical factors, where significance was taken at *P* < 0.05.

### 2.4. Human Protein Atlas Database Analysis

To investigate the protein expression levels of the hub CDM genes involved in the signature in human normal and breast cancer tissues, we downloaded the representative images of the immunohistochemical assay from the Human Protein Atlas (http://www.proteinatlas.org) [16].

### 2.5. Immunity-Related Analysis

We further used the Tumor Immune Estimation Resource (TIMER) [17], CIBERSORT [18], QUANTISEQ [19], XCELL [20], MCP counter [21], and Estimating the Proportion of Immune and Cancer cells (EPIC) [22] to comparatively assess cell immune responses or cellular components between two different risk groups. Moreover, single-sample GSEA (ssGSEA) was utilized to compare and quantify the subgroups of the tumor-infiltrating immune cells, as well as their immunological function, in two groups with different risks. Previous research yielded a list of possible immunological checkpoints.

### 2.6. Statistical Analysis

Chi^2^ tests were used to examine relationships between categorical and continuous variables in testing and training sets from TCGA. Univariate Cox proportional hazards regression (CPHR) analysis and the Kaplan–Meier technique were used to confirm candidate CDM genes that were substantially linked to the OS of patients with breast cancer. Stepwise multivariate CPHR studies were used to identify both important clinical factors and the risk score calculation. Survival curves were produced using the Kaplan–Meier technique, which were then evaluated using the log-rank test method. A time-dependent ROC curve was used to evaluate the risk model's specificity and sensitivity in predicting patients' survival. The *t*-test was carried out to compare the differences between two risk groups in training and testing sets. R software with packages was used to conduct the statistical analyses in this research. Statistical significance was determined when the *P* value was less than 0.05.

## 3. Results

### 3.1. Differentially Expressed CDM Genes and Related Pathway Enrichment Analyses in Breast Cancer

**Figure 1:**
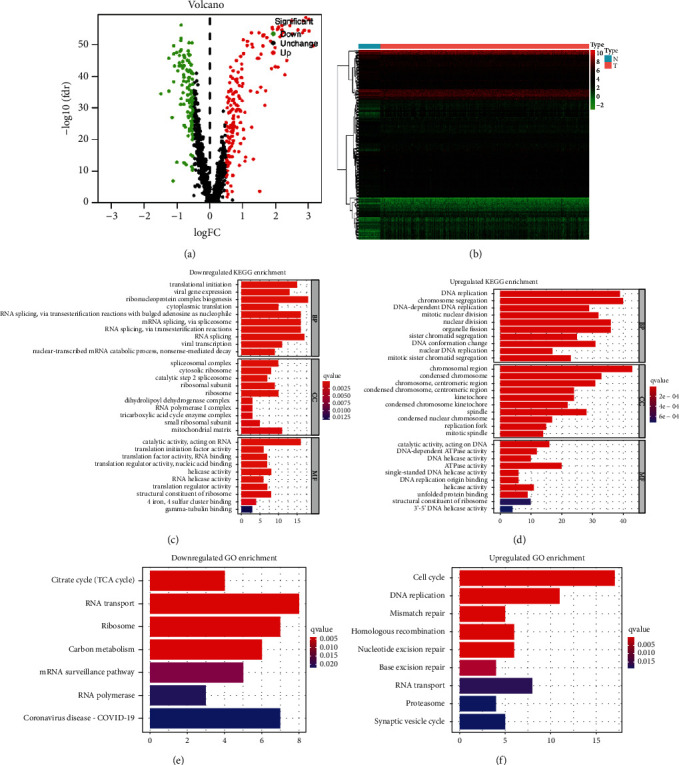
Identification of differentially expressed Cancer Dependency Map (CDM) genes in breast cancer and pathway enrichment analyses. (a) Volcano plot of CDM genes in patients with breast cancer from the TCGA database. (b) Heatmap of CDM genes. KEGG pathway enrichment of those downregulated CDM genes (c) and those upregulated ones (d). GO pathway enrichment of those downregulated CDM genes (e) and those upregulated ones (f).

We first analyzed the expression levels of 1246 CDM genes between the patients and controls to identify differentially expressed ones. As shown in Figure 1(a), a total of 290 CDM genes were chosen, which composed of 116 downregulated genes and 174 upregulated ones. The heatmap in Figure 1(b) revealed the 290 differentially expressed genes between patients and controls with FDR<0.05 and log2|fold change|>0.5. To investigate these identified CDM genes' function and related mechanisms, we carried out functional analyses of them, which mainly concluded KEGG and GO pathway enrichment analyses. As shown in Figures 1(c) and 1(d), downregulated CDM genes were enriched in RNA splicing, translational initiation, and so on, while upregulated CDM genes were enriched in DNA replication, chromosome segregation, nuclear division, and so on. GO enrichment analysis revealed that those downregulated genes mainly enriched in RNA transport and citrate cycle, and upregulated genes enriched in cell cycle, DNA replication, and homologous recombination (Figures 1(e) and 1(f)).

### 3.2. Identification of Prognostic-Related CDM Genes and Construction of the Prognostic Model

**Figure 2:**
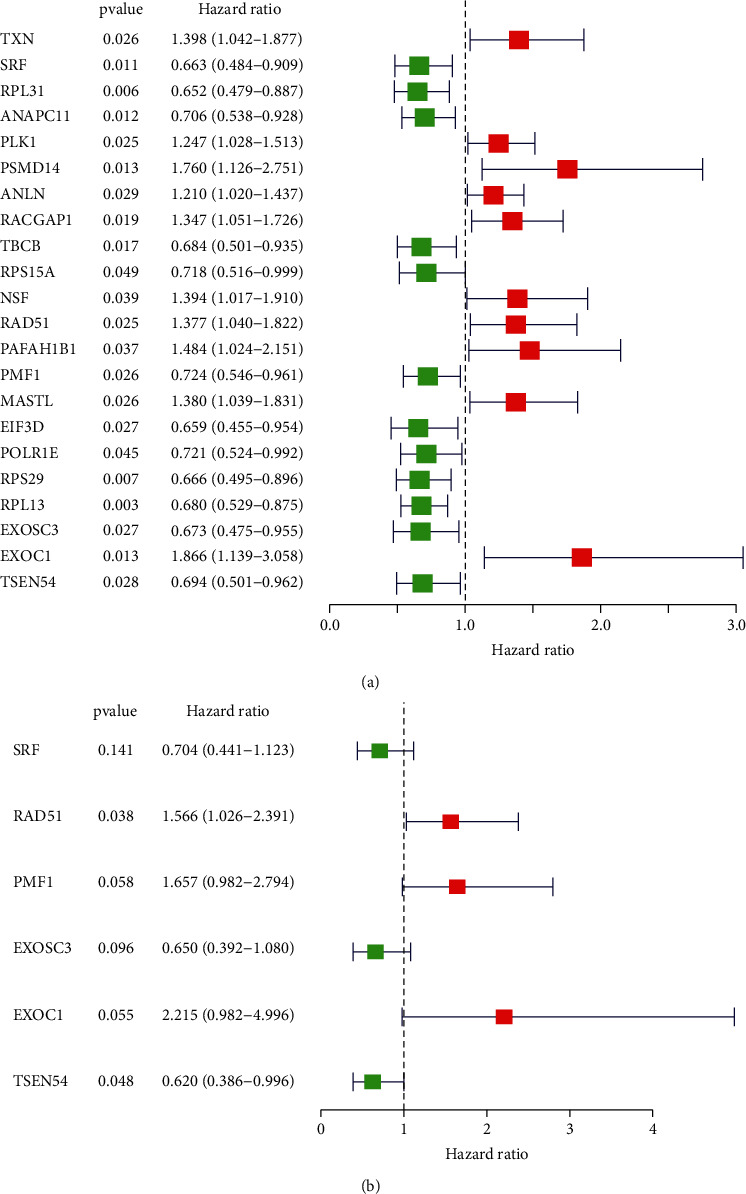
Identification of CDM genes with the predictive value and construction of risk model for breast cancer. Univariate (a) and multivariate (b) Cox regression analyses on the differentially expressed CDM genes.

To explore the effects of CDM genes in predicting the prognosis of patients with breast cancer, we studied the relationship between the 290 differentially expressed CDM genes and the OS of patients with breast cancer by univariate Cox regression analysis. As shown in Figure 2(a), 22 CDM genes were selected as candidate genes for their significantly association with the OS of breast cancer patients. Then, we further carried out the multivariate Cox analysis and identified six hub genes with an independent prognostic value (Figure 2(b)). Based on the six candidate hub genes, we established a model to predict the prognosis for those patients with breast cancer: risk score = coef (i) ∗ exp (i), where coef (i) meant the multivariate Cox regression coefficient and exp (i) meant the expression level. We assessed the risk score of each sample from the TCGA training set, and the median value was identified as the cut-off, based on which patients with breast cancer were divided into the low-risk or high-risk group.

### 3.3. Validation of the Risk Model in the TCGA and GEO Databases

**Figure 3:**
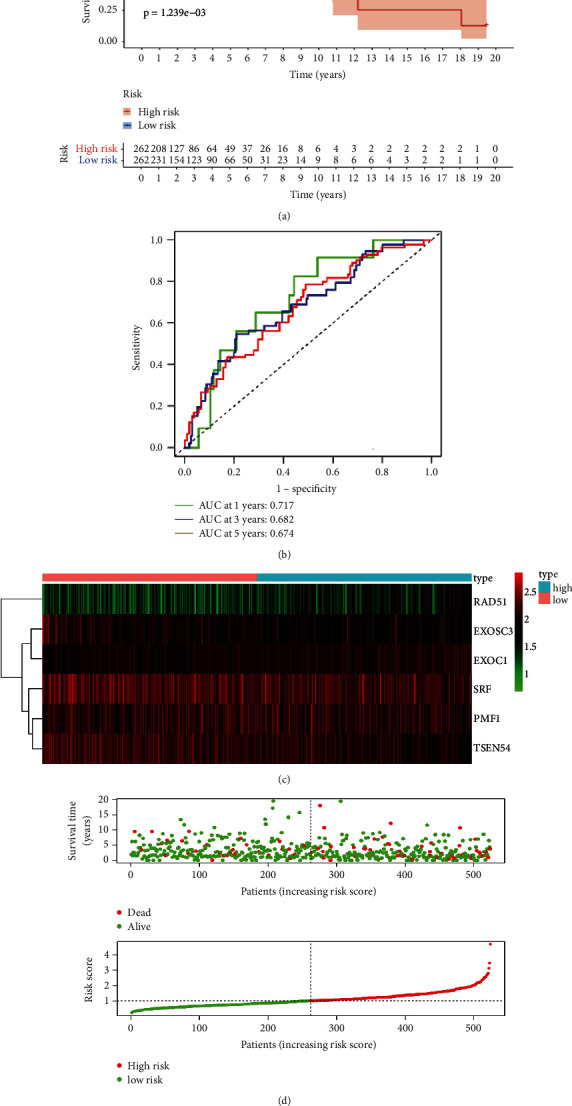
Validation of the six CDM gene-based signature in the TCGA training set. (a) The Kaplan–Meier curve for the overall survival (OS) of breast cancer samples based on the constructed signature in the TCGA training set. (b) Time-dependent receiver operating characteristic (ROC) curve analysis of the signature for predicting OS of patients with breast cancer. (c) Heatmap of the signature in the TCGA training set. (d) Risk score distribution and OS status in the TCGA training set.

**Figure 4:**
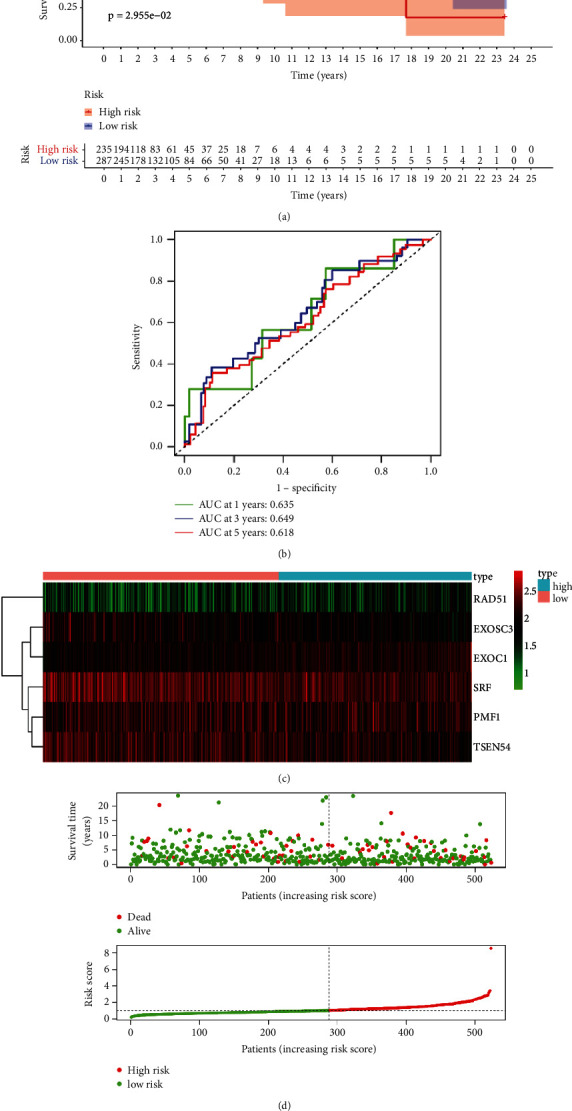
Validation of the six CDM gene-based signature in the TCGA testing set. (a) Kaplan–Meier curves for OS of patients with breast cancer in the TCGA testing set. (b) Time-dependent ROC curve analysis for predicting OS of breast cancer samples. (c) Heatmap of the signature in the TCGA testing set. (d) Risk score distribution and OS status in the TCGA testing set.

To evaluate the prognostic value of the constructed risk model, we performed Kaplan–Meier survival analysis and found that patients with high risk in the training set had poorer prognosis than those with low risk (Figure 3(a)). The time-dependent ROC analysis showed the area under the ROC curve (AUC) was 0.717 at 1 year (Figure 3(b)), which indicated the good prognostic value of the identified model in the TCGA training set. The heatmap of the hub 6 CDM genes and patients' survival status in the training set are shown in Figures 3(c) and 3(d). To further explore the predictive value of the identified risk model composed of 6 CDM genes in the TCGA testing set, we carried out survival analysis and ROC analysis. As shown in Figure 4(a), patients with lower risk scores in the testing set had significantly longer survival time than those with higher risk scores. Also, the AUC was 0.635 at 1 year in the testing set (Figure 4(b)). The survival status and risk scores of patients in the testing set, along with the expression heatmap of the 6 hub genes in testing set are shown in Figures 4(c) and 4(d).

Further validation of the risk model on the patients with breast cancer by GSE53031 cohort from the GEO database were carried out, and the results (Figure S1) showed that the six identified CDM genes were closely linked to the prognosis of patients not only from TCGA but also from an external dataset from GEO. To further validate the efficiency of the signature based on CDM genes in predicting the survival, we compared the constructed signature with another two signatures reported recently in the TCGA-breast cancer cohort. The nine-gene signature derived from Wang's research (WangSig) was built based on the analyses of the ferroptosis-related information from breast cancer [23]. However, the six-gene signature from Mo's study (MoSig) was constructed based on integrated multiomics data analysis [24]. As shown in Figure S2, CDMSig had a higher AUC value (AUC = 0.684) than that of the WangSig (AUC = 0.587) and the MoSig (AUC = 0.478), suggesting that the CDM gene-based signature we built had a good predictive performance.

### 3.4. Establishment of a Nomogram Combining with Clinical Characteristics

**Figure 5:**
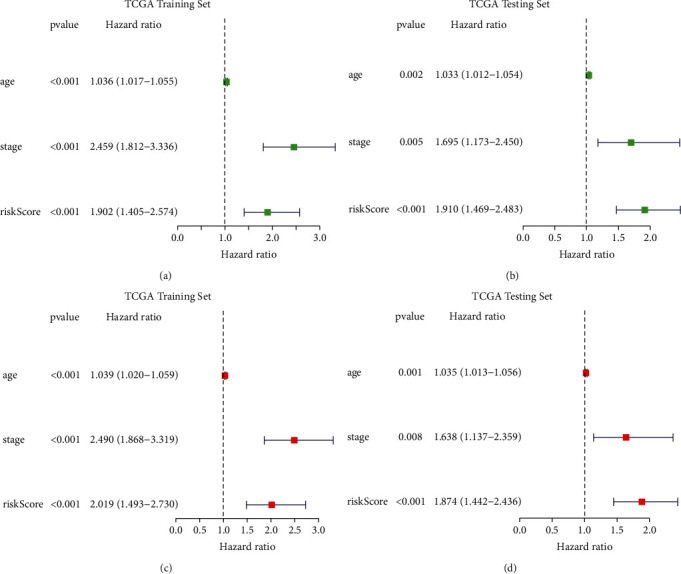
Validation for the prognostic value of the constructed risk model along with different clinical characteristics. Univariate (a) and multivariate (c) COX regression analysis of the signature and different clinical features in the TCGA training set. Univariate (b) and multivariate (d) COX regression analysis of the signature and different clinical features in the TCGA testing set.

**Figure 6:**
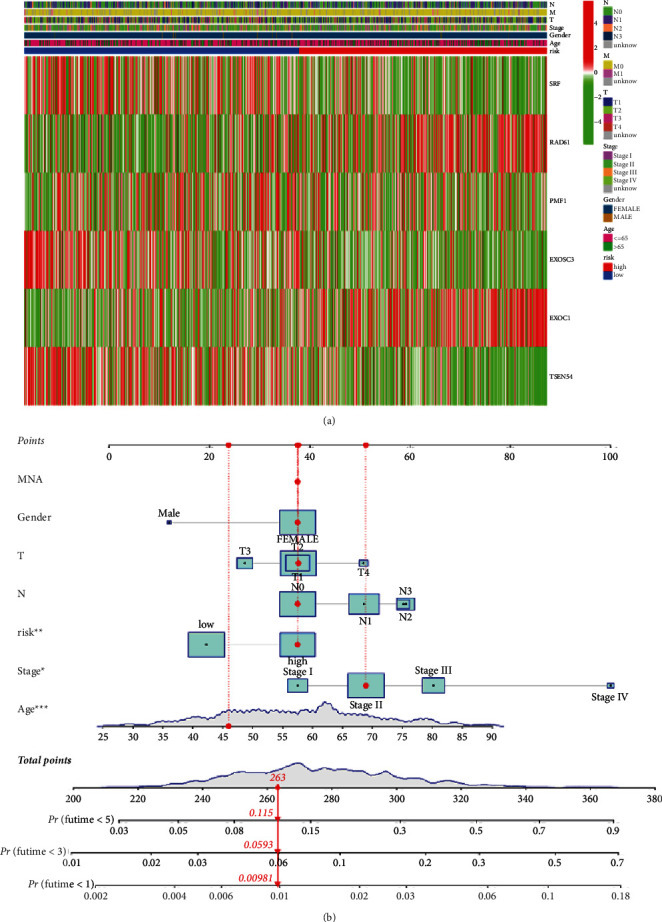
Construction of a nomogram for prediction. (a) Heatmap for the CDM gene-based signature with clinicopathological manifestations in TCGA-breast cancer set. (b) Nomogram for predicting 1-, 3-, and 5-year OS of patients with breast cancer.

Considering that the constructed risk model was significantly associated with poor prognosis, we combined the OS of breast cancer patients with their clinical features and performed univariate and multivariate Cox analysis to explore whether the signature we constructed could serve as an independent factor to predict the prognosis. As shown in Figure 5, all the *P* values were less than 0.001, indicating that the signature had an independent prognostic value. The heatmap in Figure 6(a) showed the association between the six identified CDM genes in the prognostic risk model and clinical manifestations of all breast cancer samples from TCGA. Then, by combing all these clinical features and the constructed signature, we established a nomogram to expand the application and availability of the constructed risk model in clinic (Figure 6(b)).

### 3.5. Protein Expression Levels of Identified Hub CDM Genes

**Figure 7:**
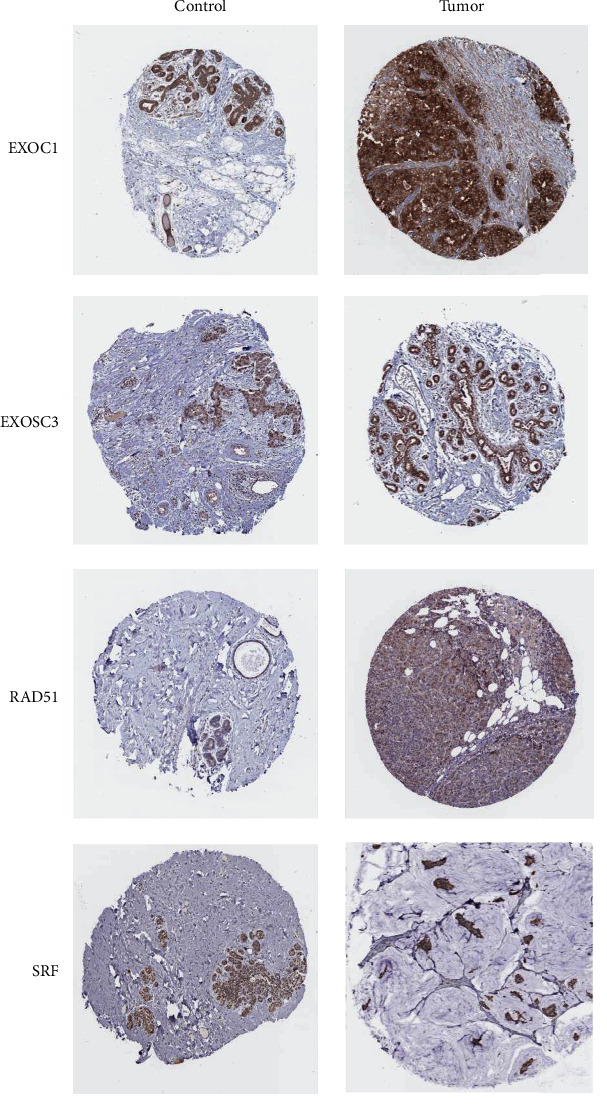
The representative images of the immunohistochemical images of identified CDM genes from the Human Protein Atlas database.

We used the Human Protein Atlas online to evaluate the protein expression levels of the 6 CDM genes in breast cancer sample and normal human mammary tissue. As shown in Figure 7, SRF and EXOSC3 expression levels were lower in tumor tissues than in control tissues, while RAD51 and EXOC1 were significantly higher in tumor tissues. These results indicated that RAD51 and EXOC1 might be pathogenic factors, while SRF and EXOSC3 might be protective factors in breast cancer, which were consistent with their coefficients in the risk model.

### 3.6. Immune-Related Analysis of the Established Risk Model

**Figure 8:**
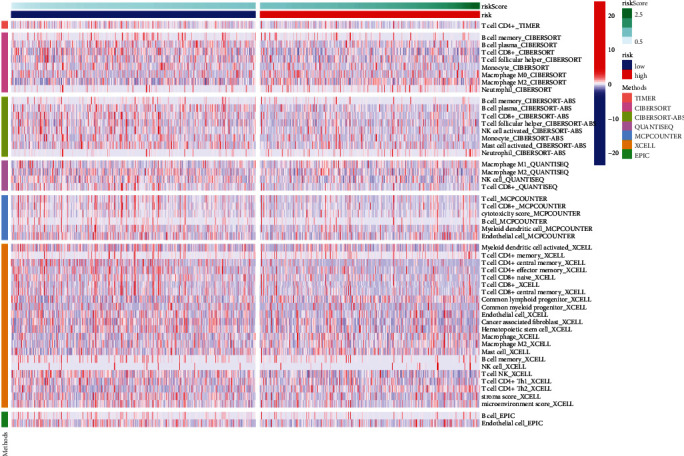
A heatmap of immune responses according to TIMER algorithms, CIBERSORT, QUANTISEQ, XCELL, MCP counter, and EPIC between the high- and low-risk groups of breast cancer.

**Figure 9:**
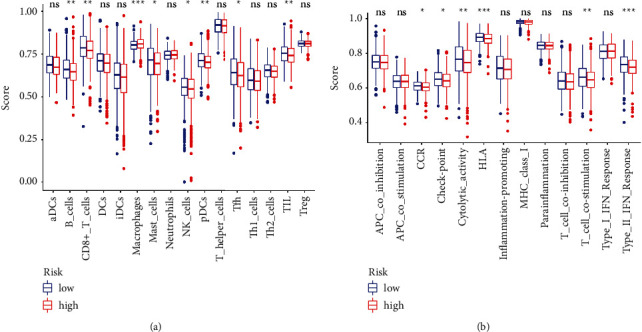
Immune-related analyses for the patients with breast cancer from TCGA. (a) Correlation analysis for immune cell subpopulations between the two breast cancer risk groups. (b) ssGSEA for the association between immune cell subpopulations and related functions between two risk groups.

**Figure 10:**
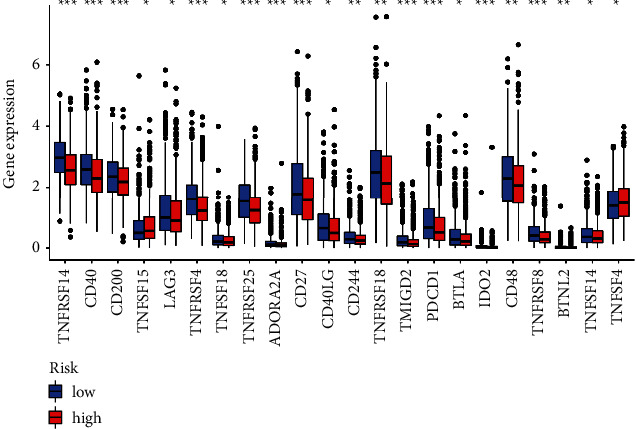
The expression levels of immune checkpoint genes between the two risk groups.

As the immune status has been reported significantly associated with breast cancer [25], we further explored the immune responses between two risk groups via TIMER algorithms, CIBERSORT, QUANTISEQ, XCELL, MCP counter, and EPIC (Figure 8). The enrichment fractions of immune cells were analyzed to explore the association between the immune status and risk scores in patients with breast cancer from the TCGA database. As shown in Figure 9, the antigen presentation process contents, including HLA, B cells, NK cells, and mast cells, were significantly declined in the high-risk group. Also, comparing with the patients in the low-risk group, the scores of Tfh cells, T cell co-stimulation, and CD8+ T cells were obviously lower in the patients with higher risk scores, which indicated the difference in the regulation of T cells between the two risk groups. In addition, the fractions of checkpoints, CCR, and the activity of type II IFN response were lower in patients in the high-risk group, while the macrophages scores showed the opposite. Furthermore, we explored the expression levels of immune checkpoints and found that LAG3, CD27, PDCD1, CD40, and TNFRSF14 were significantly different between two groups with different risk scores (Figure 10).

## 4. Discussion

It still remains difficult for us to identify genes whether they are essential for the survival of tumor, for that most tumors especially malignant ones are closely associated with genetic mutations. These genetic mutations are often related with the cell growth and specific vulnerabilities to specific damages [26]. It has been reported that some of these mutations have potential as therapeutic targets [27]. Hence, the challenge nowadays for us is how to identify specific targetable vulnerabilities for every cancer by using current tools [28]. Scientists have profiled about hundreds of cell models to elucidate genomic information. Large-scale databases have also been searched for hoping that the identification of genetic targets would help a lot in developing novel therapies, identifying the certain treatments for patients and allowing physicians to better understand the weaknesses of cancers. Actually, a project named Cancer Dependency Map, has committed to remain freely available to the public. The related information could be downloaded online freely (https://depmap.org/portal/depmap/). CDM has been used as a novel method to explore the priority targets, as well as the drug sensitivity to different cancers. Hence, it will help scientists discover new targeted therapies as soon as possible, thus facilitating progress in precision therapy [29].

To date, studies on the roles of CDM genes are scarce [30, 31]. In this study, 1246 CDM genes were analyzed and a total of 290 differentially expressed CDM genes had been identified. The univariate and multivariate Cox regression analysis revealed that six CDM genes (SRF, RAD51, PMF1, EXOSC3, EXOC1 and TSEN54) were associated with the survival of patients with breast cancer. Hence, a CDM gene-related risk model was established in the training set based on the six genes and was further validated in TCGA testing set. It was further proven by ROC analysis to be reliable in predicting the prognosis of breast cancer patients. In addition, we constructed a nomogram to improve clinical practicality of the constructed risk model for patients with breast cancer. These results might make it easier for us to understand the mechanisms of breast cancer and help us explore new biomarkers for patients with breast cancer. Functional and enrichment pathway analyses revealed that upregulated CDM genes mainly took part in DNA replication, nuclear division, chromosome segregation, cell cycle, DNA replication, and homologous recombination, while downregulated CDM genes were mainly involved in RNA splicing, translational initiation, RNA transport, and citrate cycle. During the past few years, it has been reported that abnormal metabolism [32–34] and processing [35–37] of DNA play critical roles in many neoplastic diseases.

The results of the multivariate Cox regression analysis of the six identified CDM genes revealed that all of them had prognostic value for breast cancer. Consistent with our findings, He et al. [38] demonstrated that SRF could regulate the promoter activity of HOTAIR, a negative prognostic factor for breast cancer, thus affecting the invasiveness and proliferation of breast cancer cells. The overexpression of RAD51 has been found in various cancers, including breast cancer, and it is revealed closely associated with poor survival [39]. Also, by combining with Nrf-2, PMF1 could regulate the expression of SSAT [40], which affects the apoptotic cell death in breast cancer cells [41]. Although there has been few researches on the roles of EXOSC3 and EXOC1 in breast cancer, the results from the Human Protein Atlas (Figure 8) showed that the expression of EXOSC3 and EXOC1 were significantly different between tumor tissues and control tissues, and their expression levels showed the same trend with the results of multivariate Cox regression analysis.

As breast cancer is characterized by tumor heterogeneity [42, 43], which is also the main reason that leads to the complexity of treatment [44], a more reliable model with prediction value is urgently needed. Although there have been several signatures constructed for predicting the prognosis of patients with breast cancer [45–47], the relationship between these indicators and breast cancer remains unclear. Then, using the six identified CDM genes, we established a risk model with prognostic value for patients with breast cancer by combining the data from the TCGA training set. The ROC analysis demonstrated that the constructed signature could select out patients with poor prognosis by calculating their values. The ROC analysis based on data from the TCGA testing set showed the same results. Further confirmation of the reliability of the constructed signature was done by Kaplan–Meier analysis. What is more, the multiple Cox regression analyses in the TCGA training set and testing set both revealed that the CDM gene-based signature could serve as an independent indicator for predicting the prognoses of patients with breast cancer. All of the findings aforementioned pointed out the CDM gene-based prediction risk model had clinical practicality. A nomogram was further constructed to provide an easier method to enable the physicians predict the survival of patients with breast cancer.

It is well known that in the pathogenesis of breast cancer, tumor-infiltrating immune cells play important roles [48] since over 70% of patients with breast cancer contain tumor-infiltrating lymphocytes [49]. To investigate the relationship of the constructed risk model and the immune status of patients with breast cancer, we divided all samples from TCGA into two risk groups according to their scores. Of note, consistent with previous researches, immune checkpoint genes, CD40, and TNFRSF14 were negatively related with the CDM gene-based signature. The activation of CD40 is important in the generation of T cell immunity [50]. Besides, the absence of TNFRSF14 would change the microenvironment of tumors [51, 52].

However, this study also has limitations. First, the information from TCGA database did not contain information of the preoperative and postoperative parameters, which made it impossible to carry out more comprehensive analyses with these potential and important factors. Second, since the data of this study were downloaded from TCGA, it was more in line with the characteristics of a retrospective study. Hence, to further validate the results, we need to carry out prospective clinical studies.

In summary, we explored the potential functions of the differentially expressed CDM genes in patients with breast cancer. A CDM gene-based risk model was constructed, which could predict the prognoses of patients with breast cancer. Besides, a clinically practical nomogram that can predict prognoses of breast cancer patients was constructed to help individual management in clinic.

## Data Availability

The datasets generated and analyzed during the current study are available in TCGA (http://cancergenome.nih.gov/abouttcga) and GEO (https://www.ncbi.nlm.nih.gov/geo). The authors acknowledge TCGA and GEO database for providing the platform for uploading the meaningful datasets.

## Supplementary Materials

Supplementary Materials Supplementary File Figure S1: validation of the six CDM gene-based signature in an external dataset from GEO database. (A) Kaplan–Meier curves for OS of patients with breast cancer in GEO cohort. (B) Time-heatmap of the signature in GEO dataset, along with the risk score distribution and OS status. Supplementary File Figure S2: time-dependent ROC curves study of 3-year OS for the CDMSig, WangSig, and MoSig.
