# Supplementary figures and images for: Construction and Validation of a Newly Prognostic Signature for CRISPR-Cas9-Based Cancer Dependency Map Genes in Breast Cancer
Source: J Oncol. 2022 Jan 19;2022:4566577. doi: 10.1155/2022/4566577 (PMC8791742; doi:10.1155/2022/4566577)

**Supplementary Figure 1**


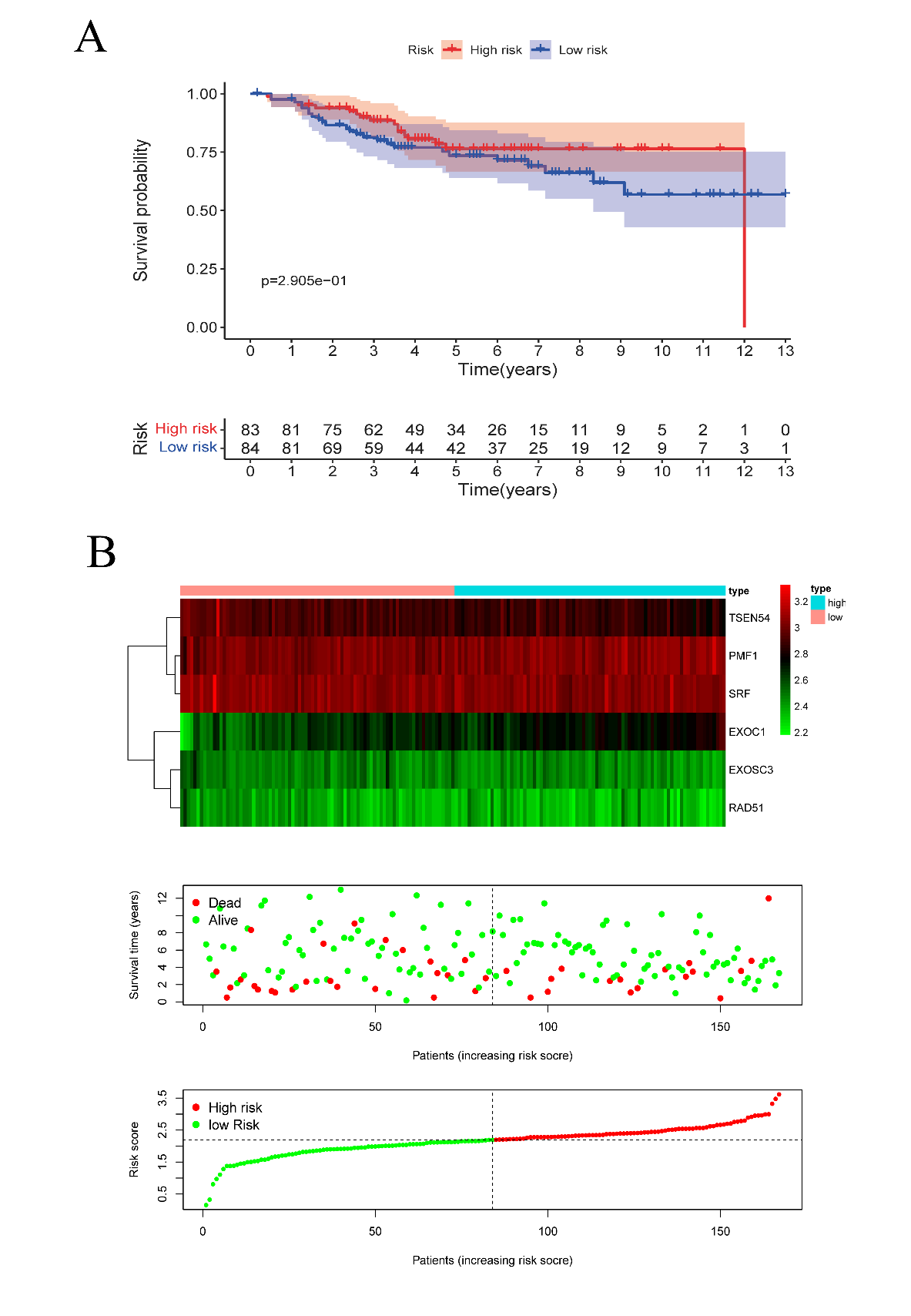


**Supplementary Figure 2**


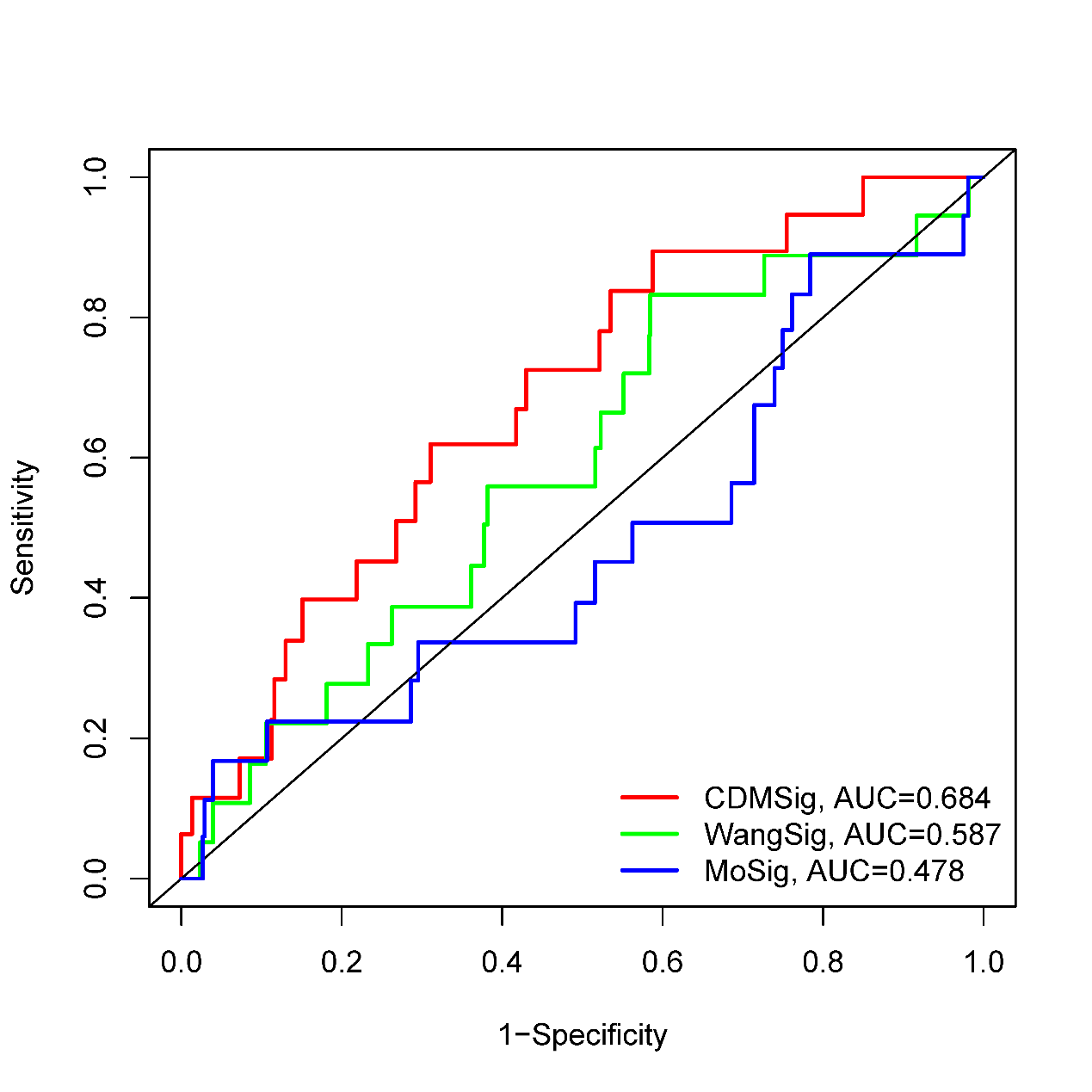

Supplement: Supplementary Materials — Supplementary File Figure S1: validation of the six CDM gene-based signature in an external dataset from GEO database. (A) Kaplan–Meier curves for OS of patients with breast cancer in GEO cohort. (B) Time-heatmap of the signature in GEO dataset, along with the risk score distribution and OS status. Supplementary File Figure S2: time-dependent ROC curves study of 3-year OS for the CDMSig, WangSig, and MoSig. [file 4566577.f1.docx]
